# Design of a peptidic inhibitor that targets the dimer interface of a prototypic galectin

**DOI:** 10.18632/oncotarget.5403

**Published:** 2015-10-01

**Authors:** Maria Claudia Vladoiu, Marilyne Labrie, Myriam Létourneau, Philippe Egesborg, Donald Gagné, Étienne Billard, Andrée-Anne Grosset, Nicolas Doucet, David Chatenet, Yves St-Pierre

**Affiliations:** ^1^ INRS-Institut Armand-Frappier, Université du Québec, Laval, Québec, H7V 1B7 Canada

**Keywords:** galectin, inhibitor, peptide, apoptosis, T-cells

## Abstract

Galectins are small soluble lectins that bind β-galactosides via their carbohydrate recognition domain (CRD). Their ability to dimerize is critical for the crosslinking of glycoprotein receptors and subsequent cellular signaling. This is particularly important in their immunomodulatory role via the induction of T-cell apoptosis. Because galectins play a central role in many pathologies, including cancer, they represent valuable therapeutic targets. At present, most inhibitors have been directed towards the CRD, a challenging task in terms of specificity given the high structural homology of the CRD among galectins. Such inhibitors are not effective at targeting CRD-independent functions of galectins. Here, we report a new class of galectin inhibitors that specifically binds human galectin-7 (hGal-7), disrupts the formation of homodimers, and inhibits the pro-apoptotic activity of hGal-7 on Jurkat T cells. In addition to representing a new means to achieve specificity when targeting galectins, such inhibitors provide a promising alternative to more conventional galectin inhibitors that target the CRD with soluble glycans or other small molecular weight allosteric inhibitors.

## INTRODUCTION

Cancer is a complex pathology manifested by uncontrolled growth of cells that have undergone various transformations from physiologically normal cells. Several hallmarks provide a methodical and rational approach in studying this disease, namely the sustaining of proliferative signaling, evasion of growth suppressors, resistance to cell death, replicative immortality, angiogenesis, activation of invasion, and metastasis [1]. In recent years, however, strong evidence has highlighted the critical roles of immune cells present in the tumor micro-environment [2, 3]. For instance, one way that tumor cells can modulate and escape immune destruction is by secretion of various factors such as pro-inflammatory eicosanoids, cytokines, chemokines and other soluble signaling molecules leading to the formation of an immunosuppressive tumor micro-environment [4].

Galectins are multifunctional proteins belonging to the animal lectin family. All galectins share similar binding affinities to β-galactosides and display high amino acid sequence homology among their carbohydrate-binding domains (CRDs) [5]. Fifteen different members have been identified and divided in three sub-groups according to their structure: prototype galectins containing one CRD (Gal-1, -2, -5, -7, -10, -13, -14 and -15), tandem-repeat galectins containing two-CRDs covalently linked (Gal-4, -6, -8, -9 and -12) and a chimera-type galectin containing multiple CRDs linked by their amino-terminal domain (Gal-3) [6]. While these proteins perform homeostatic functions inside normal cells, under pathological or stress conditions, cytosolic galectins are released either passively from dead cells or actively *via* non-classical secretion pathways [7]. Once in the extracellular milieu, they bind all glycosylated growth receptors on the surface of normal and cancer cells to set their signaling threshold [8, 9]. Such properties enable galectins to kill infiltrating immune cells while promoting growth of tumour cells [9]. Galectins are thus ideal targets for effective therapeutics, and new approaches are therefore being developed to modulate their activities [10]. These avenues have focused mainly on carbohydrate-based inhibitors disrupting extracellular galectins, which form multivalent complexes with cell surface glycoconjugates to deliver CRD-dependent intracellular signals that modulate cell activation and survival/apoptosis. Despite decades of research, however, the progression in this field has been very slow. In most cases, these inhibitors are high molecular weight, naturally occurring polysaccharides that are used to specifically block the binding of extracellular galectins to carbohydrate structures [11–14]. Unfortunately, such inhibitors often display low affinity, lack of selectivity for a given galectin due to highly conserved homology among galectin CRDs, and are not effective at targeting CRD-independent functions of galectins. Indeed, several studies have shown that several critical biological processes of galectins are mediated *via* CRD-independent interactions [15–18].

**Figure 1:**
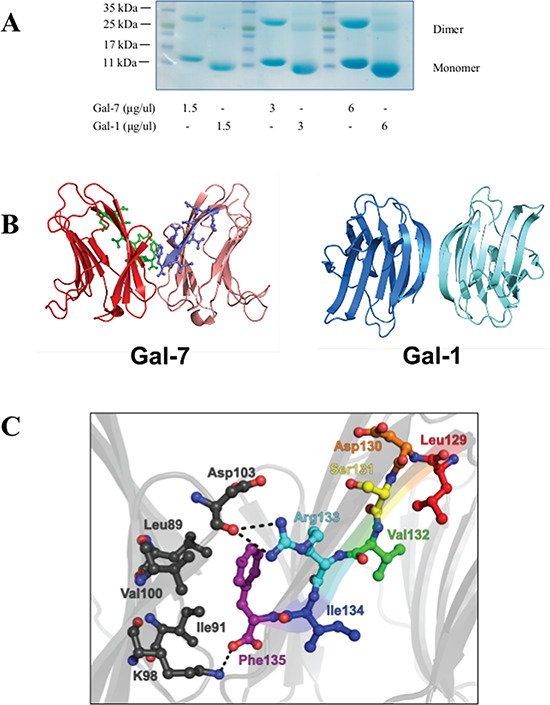
The dimeric structure of hGal-7 **A.** Dimer formation of recombinant hGal-7 and hGal-1 at increasing concentrations were compared by polyacrylamide gel electrophoresis in native conditions. **B.** Structural representation of the hGal-7 (PDB 1BKZ) and hGal-1 (PDB 3W58) dimers with residues 129–135 colored in green and magenta on the hGal-7 dimer interface. Dimer formation in hGal-7 proceeds through a “back-to-back” topology of the monomers while hGal-1 adopts a “side-by-side” structural arrangement, affording additional specificity for galectin inhibition. **C.** Molecular interactions implicated in the wild-type hGal-7 dimer interface between residues 129–135 of the first hGal-7 monomer (in various colors) and facing residues on the second hGal-7 monomer (in black) (PDB 1BKZ). Hydrogen bonding and electrostatic interactions are identified as dashed lines. The side chain of Phe135 is also involved in a number of van der Waals interactions [29]. The structures were prepared with PyMOL.

Sequencing of galectins isolated from amphibians, birds, fish, and mammals has revealed extensive sequence similarity [19, 20]. In addition to the presence of a CRD, all galectins harbor a highly conserved three-dimensional structure characterized by a jelly-roll topology composed of an 11- or 12-strand anti-parallel β-sandwich of approximately 135–140 amino acid residues [21]. One of the most common and important structural features associated with galectin function is their ability to form homodimers (Fig. 1B). This is particularly true for the prototype galectins, which consist of two ~14–15 kDa subunits that are non-covalently associated in a monomer-dimer equilibrium [22]. Studies of ancestral structures of fish galectins have indeed shown that galectins have gone through selective pressure for stabilizing this homodimer structure to increase their affinity for their ligand(s) [23]. Such multivalency is critical for galectins to trigger intracellular signaling following their binding to cell surface receptors [24–26]. In the present work, we report a novel peptide-based galectin inhibitor that was specifically designed to disrupt the formation of galectin-7 dimers and its pro-apoptotic function.

## RESULTS

As depicted with G protein-coupled receptors, peptides derived from the dimeric interface were shown to disrupt GPCR dimers by interfering with critical interactions between amino acids located at the dimer interface [27, 28]. We hypothesized that the ability of hGal-7 to form homodimers is mediated by critical residues located at the homodimer interface located in a distant region of the CRD. Using a previously described dimeric crystal structure of hGal-7 [29], critical residues possibly involved in the formation of the dimer interface were identified based on their propensity to form hydrogen bonding, hydrophobic, or van der Waals interactions between both protomers of the complex (Fig. 1B). In addition, the design of our peptides was also based on the structural analysis of the hGal-7 dimeric interface, recently reported by Ermakova and colleagues [30]. Specifically, their analysis revealed that certain residues were likely to be involved in the dimerization of hGal-7, most notably residues R14, R20, R22, E87, L89, D94, D95, D103, A104, Q105, D130 and F135. These residues appear to symmetrically associate with their respective partner on the second monomer through H-bonding, electrostatic or hydrophobic interactions, or *via* the formation of disulfide bridges. As such, the peptides were designed as to rationally mimic and disrupt the hGal-7 segment between residues 13–25 and 129–135, since those residues appear to be directly involved in the stabilization of the dimeric structure (Fig. 1C) and consequently hindering the interaction of R14, R20, R22, D130 and F135 with their mirror partners. Accordingly, peptides corresponding to these regions were synthesized, *i.e*. hGal-7_(13–25)_ (H-Ile-Arg-Pro-Gly-Thr-Val-Leu-Arg-Ile-Arg-Gly-Leu-Val-NH_2_) and hGal-7_(129–135)_ (H-Leu-Asp-Ser-Val-Arg-Ile-Pro-NH_2_). To determine whether these peptides could inhibit the formation of the hGal-7 homodimer, we incubated 0.5 μM of recombinant hGal-7 with increasing concentrations of hGal-7_(13–25)_ or hGal-7_(129–135)_ and measured the formation of homodimers.

**Figure 2:**
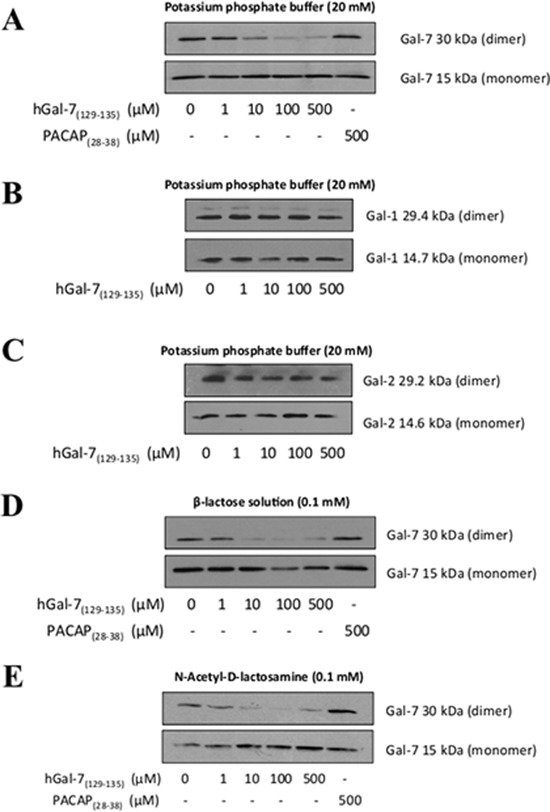
Disruption of the hGal-7 dimer due to increasing concentrations of hGal-7(129–135) **A.** The recombinant hGal-7 (0.5 μM) was incubated with increasing concentrations of hGal-7_(129–135)_ in 20 mM potassium phosphate buffer (pH 7.1). Incubation of the recombinant **B.** hGal-1 and **C.** hGal-2 with hGal-7_(129–135)_ was performed in the same potassium phosphate buffer. The effect of hGal-7_(129–135)_ on the monomeric and dimeric forms of hGal-7/hGal-1/hGal-2 was assessed by Western blotting in native conditions with respective antibodies. The hGal-1/hGal-2 films were overexposed. PACAP_28–38_ was the control peptide used in order to ensure the specificity of hGal-7_(129–135)_. Recombinant hGal-7 (0.5 μM) was also incubated with increasing concentrations of hGal-7_(129–135)_ in 0.1 mM **D.** lactose or **E.** LacNac solutions. Results are representative of three independent experiments.

**Figure 3:**
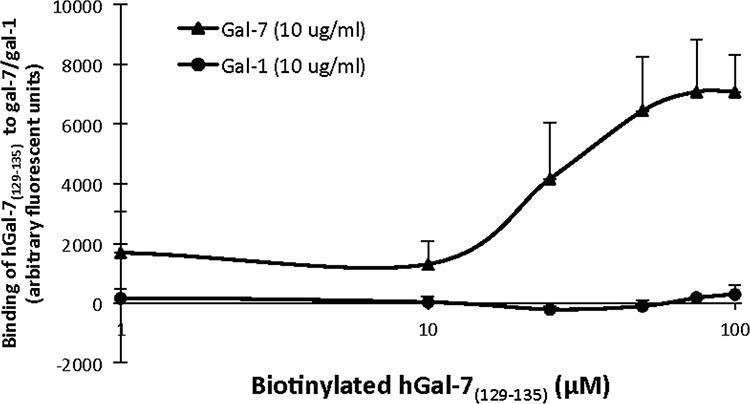
Biotin-labeled hGal-7(129–135) is capable of binding to recombinant hGal-7 Binding curve showing a dose-dependent interaction between biotin-labeled hGal-7_(129–135)_ and hGal-7 or hGal-1. Recombinant hGal-7 or hGal-1 (10 μg/ml) were coated on 96-well plates overnight and then incubated 60 min with unlabeled hGal-7_(129–135)_ (1 mM) to eliminate non-specific binding. Incubation with increasing concentrations of biotin-labeled hGal-7_(129–135)_ was performed for 120 min. Results are representative of three independent experiments. Error bars represent standard deviation.

To measure the ability of the peptides to disrupt the formation of hGal-7 dimers, we used mild denaturing native gel electrophoresis, a commonly used approach to visualize monomer-dimer equilibrium (Fig. 1A and Supplementary Fig. S1) [31–35]. Our results showed a consistent decrease of hGal-7 homodimers starting at a 10 μM concentration of hGal-7_(129–135)_, with a saturation dose of 100 μM (Fig. 2A). Similar results were obtained with hGal-7_(13–25)_ but this compound appeared less potent than hGal-7_(129–135)_ to disrupt hGal-7 homodimers and was consequently not used in subsequent experiments (*data not shown*). No such effect was observed using the control pituitary adenylate cyclase-activating polypeptide (PACAP) peptide, which was selected based on similarity in amino acid length and minimal toxicity on the cell line, PACAP(28–38) (Fig. 2A), or on a recombinant human gal-1 (hGal-1) or gal-2 (hGal-2) (Fig. 2B, 2C). hGal-1 and gal-2 were chosen as galectin selectivity controls since they are protype galectin that share the greatest sequence similarity to galectin-7 (38%) [29]. Moreover, the ability of the hGal-7_(129–135)_ peptide to disrupt the formation of hGal-7 homodimers was not inhibited by the presence of lactose or LacNac (Fig. 2D, 2E). Further, the ability of hGal-7_(129–135)_ to bind hGal-7 in a concentration-dependent and specific manner was further confirmed using a solid phase binding assay. In this assay, a biotinylated version of hGal-7_(129–135)_ still capable of specifically inhibiting the formation of hGal-7 homodimers (Supplementary Fig. S2) was used to measure binding on immobilized recombinant hGal-7 (Fig. 3). Again, binding was shown to be specific since biotinylated hGal-7_(129–135)_ could bind hGal-7 and not hGal-1 or hGal-2 (Supplementary Fig. S2). This specificity at disrupting hGal-7 dimer formation is provided by distinct three-dimensional arrangements between otherwise very similar galectin homologues. Indeed, while all monomeric galectins reveal identical topologies, dimer formation in hGal-7 proceeds through a “back-to-back” topology of the monomers while other galectins, including hGal-1 and hGal-2, adopt a “side-by-side” structural arrangement (Fig. 1B) [29]. This structural organization provides additional means to specifically target and disrupt galectin function.

**Figure 4:**
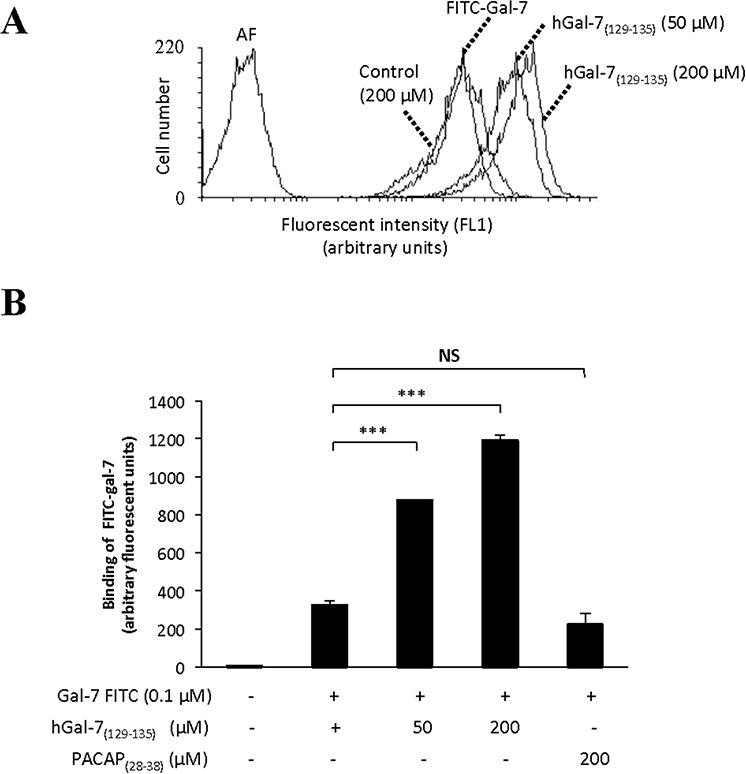
Increased binding of hGal-7 on Jurkat T cells due to increasing concentrations of hGal-7(129–135) **A.** Histogram showing the mean fluorescence intensities (MFI) of cells following binding of fluorescein isothiocyanate (FITC)-labeled hGal-7 on the surface of Jurkat T cells. **B.** Binding of FITC-labeled hGal-7 (0.1 μM) on Jurkat T cells following pre-incubation with increasing concentrations of hGal-7_(129–135)_. The PACAP_(28–38)_ peptide was used as a control. Results are representative of three independent experiments. ***p ≤ 0.05. Error bars represent standard deviations.

**Figure 5:**
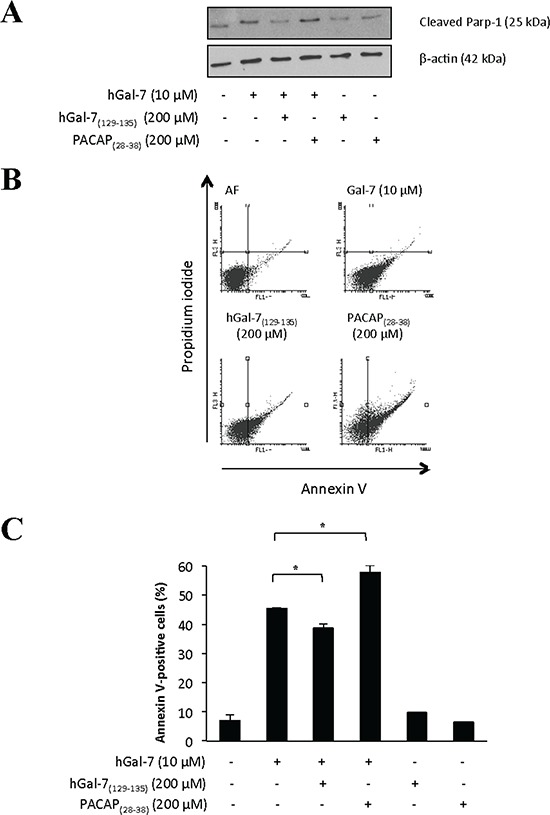
Apoptotic levels of Jurkat T cells induced by hGal-7 were decreased due to the presence of hGal-7 (129–135) **A.** Recombinant hGal-7 was pre-incubated with the respective peptide concentrations prior to its addition to Jurkat T cells for 4 h a 37°C in serum-free RPMI. Apoptosis was measured by Western blot analysis of PARP-1 cleavage. The PACAP_28–38_ was used as a control. β-actin was used as a loading control. **B.** Flow cytometric analysis of Jurkat T cells with stained Annexin V (FL1) and propidium iodide-PI (FL3) following binding of recombinant hGal-7 with or without hGal-7_(129–135)_. Cells in the lower right quadrant are representative of annexin V-positive/PI-negative, or early apoptotic cells. Cells in the upper right quadrant indicate annexin V-positive/PI-positive, or late apoptotic cells. **C.** Histogram showing the percentages of annexin V-positive Jurkat T cells obtained by adding the percentages of cells found in the lower and upper right quadrants. Results are representative of three independent experiments. Error bars represent standard deviation.

Galectins are well known for their ability to bind glycosylated cell surface receptors, most notably on Jurkat T cells, on which galectin binding induces apoptosis [36–42]. We thus tested whether hGal-7_(129–135)_ could modulate the binding of hGal-7 on Jurkat T cells, a cell model that is routinely used to test the pro-apoptotic activity of galectins [43–45]. For this purpose, recombinant hGal-7 was labeled with fluorescein isothiocyante (FITC) and its binding on the surface of Jurkat T cells was measured by flow cytometry in absence or presence of increasing concentrations of hGal-7_(129–135)_. Our results showed that hGal-7_(129–135)_ increased the fluorescent intensity of Jurkat T cells in a concentration-dependent manner following incubation with equal amounts (0.1 μM) of FITC-labeled hGal-7, as compared to fluorescence measured in absence of peptide (Fig. 4A). No such effect was observed in presence of a high concentration of the control peptide (PACAP_(28–38)_). The effect of hGal-7_(129–135)_ was specific, statistically significant (Fig. 4B), and consistent with the increased number of monomers, which bind to surface glycosylated receptors through their CRDs [38]. The hGal-7_(129–135)_ peptide also inhibited the ability of hGal-7 to induce apoptosis in Jurkat T cells, as measured by poly [ADP-ribose] polymerase 1 (PARP-1) cleavage (Fig. 5A). This inhibitory effect was dose-dependent and also observed using human peripheral blood monocytes (Supplementary Fig. S3). No such effect was observed with the control peptide. This effect on apoptosis was confirmed by flow cytomtery using Annexin V/propidium iodide (PI) staining (Fig. 5B and 5C). Incubation of hGal-7_(129–135)_ (or PACAP_(28–38)_) alone did not induce apoptosis in Jurkat T cells (Supplementary Fig. S4).

## DISCUSSION

In the present work, we report a new peptidic inhibitor that specifically binds hGal-7, disrupts the formation of homodimers, and inhibits the pro-apoptotic activity of hGal-7 on Jurkat T cells. These results show that the dimer interface of the prototype galectins is a viable option for the inhibition of galectins and a potential therapeutic target for the treatment of many diseases in which galectins are involved. To our knowledge, there have been no published reports of dimerization-disrupting inhibitors of galectins. Such inhibitors provide a new avenue for blocking hGal-7 activity and represent attractive agents against the prototype galectins because they target a second distant site from the active CRD. They are also a complementary alternative to soluble glycans or other small molecular weight allosteric inhibitors that are currently being developed for the targeting of prototype galectins. This peptidic inhibitor also represents an additional means to achieve specificity when targeting galectins. Although soluble glycans have shown physiological responses in *in vivo* assays for various pathologies where galectins are involved, there is yet little evidence of their specificity. This may not be surprising since galectins display high homology among their CRDs and often show redundancy when binding to glycosylated moieties [29, 46]. Hence, their specific targeting remains ambiguous and challenging, and the use of peptides could improve the current library of selective galectin inhibitors.

We attempted to synthesize other peptides covering other potential sites at the dimeric interface of hGal-7. This strategy was unsuccessful since these peptides were either toxic to Jurkat T cells, showed poor solubility, or were not as effective at disrupting the dimeric interface as hGal-7_(129–135)_ (*data not shown*). Although we did find a direct interaction between hGal-7_(129–135)_ and hGal-7 through a classical solid-phase binding assay, further tests are required to determine how hGal-7_(129–135)_ binds to the critical residues located at the interface. Interestingly, our preliminary data using an alanine scan strategy has already shown that the substitution of the Asp^130^ residue by an Ala moiety completely abrogates the ability of the peptide to disrupt hGal-7 homodimers (Supplementary Table 1 and Supplementary Fig. S5). It is clear, however, that hGal-7_(129–135)_ was effective at relatively high concentrations and future modifications will be needed to improve its binding affinity. These alanine scans nevertheless suggest that binding of hGal-7_(129–135)_ to monomeric hGal-7 proceeds in a non-native fashion relative to wild-type hGal-7 dimer formation. Indeed, it is currently impossible to structurally rationalize some of the molecular effects of the alanine scan results in the context of wild-type hGal-7 dimer formation. For instance, while Asp^130^ does not participate in the formation or stabilization of the wild-type hGal-7 dimer interface (Fig. 1C), its replacement to alanine clearly shows significant alteration of the hGal-7_(129–135)_ potency (Supplementary Fig. S5). These results suggest a distinct binding mode between hGal-7_(129–135)_ and monomeric hGal-7, requiring further structural analyses.

Moreover, the modulation of hGal-7 binding on the surface of Jurkat T cells and an apoptotic response were observed in the presence of the hGal-7_(129–135)_ peptide. The increase in fluorescence was the manifestation of the increased hGal-7 binding on cell surface rather than its accumulation inside the cell, since the binding assays were performed at 0°C and in the presence of sodium azide, which would limit protein internalization [47, 48]. Whether this reflects increased binding of monomeric forms of Gal-7, however, is currently unclear and will require more experiments. Nevertheless, the increase of hGal-7 cell surface binding, seen in the presence of hGal-7_(129–135)_, is specific since the control peptide, PACAP_(28–38)_, did not display such effects. Interestingly, even though an increase of cell surface hGal-7 binding was observed, a reduction in the ability of the protein to induce apoptosis of T cells was observed. This supports the idea that the increase in hGal-7 binding on cell surface is due to the increased access of the monomer's CRDs binding glycosylated residues on cell surface receptors while lacking intracellular signaling, highlighting that effective crosslinking of cell surface receptors is critical for inducing apoptosis [25, 49]. Such peptide may thus be a valable tool to study the ability of Gal-7 to modulate T cells functions *in vitro* or *in vivo*. We and others have indeed shown that Gal-7 has a strong cytotoxic and inhibitory effects on T cell functions and survival [38, 50], consistent with the ability of galectins to induce to a local (and systemic ?) immunosuppression when produced by cancer cells. Intriguingly, a slight increase in apoptosis is observed with the control peptide, even though the PACAP_(28–38)_ peptide did not display any toxicity on Jurkat T cells alone. PACAP_(28–38)_ is a positively charged peptide that binds cell surface phospholipids but does not translocate within the cytoplasm [51]. Even if the peptide alone does not induce apoptosis, the perturbation exerted by PACAP_(28–38)_ at the cell surface might slightly increase hGal-7-induced necrosis.

## MATERIALS AND METHODS

### Cell lines and reagents

The Jurkat cell line was maintained in RPMI 1640 medium. The culture medium was supplemented with 10% [v/v] fetal bovine serum, 2 mmol/L L-glutamine, 10 mM HEPES buffer, and 1 mM sodium pyruvate. All cell culture products were purchased from Life Technologies (Burlington, ON, Canada). Isolation of peripheral mononuclear cell (PBMCs) was performed as previously described [17].

### Peptide synthesis

The hGal-7_(129–135)_ peptide and its Ala-substituted derivatives ([Ala^130^]hGal-7_(129–135)_, [Ala^131^]hGal-7_(129–135)_, [Ala^133^]hGal-7_(129–135)_ and [Ala^135^]hGal-7_(129–135)_), as well as the control peptide PACAP28–38 (an inactive fragment of the human Pituitary Adenylate Cyclase-Activating Polypeptide (PACAP)), were synthesized using standard Fmoc chemistry, as previously described [52]. Briefly, all peptides were assembled using a semi-automatic multi-reactor system. The Rink-amide resin was used as the solid support, and the amino acids of the peptide sequences were introduced under their Fmoc-N-protected form, *i.e*. 3 eq based on the original substitution of the resin (0.7 mmol.g^−1^). Couplings of the protected amino acids were mediated by (Benzotriazol-1-yloxy)tris(dimethylamino)phosphonium hexafluorophosphate (BOP, 3 eq) and N, N-Diisopropylethylamine (DIPEA, 5 eq) in DMF for 1 h. Coupling efficiency was monitored with the qualitative ninhydrin test. Fmoc removal was achieved with 20% piperidine in DMF for 20 min. In order to obtain a biotin-conjugated peptide, an ε-amino acid (Fmoc-Ahx-OH) linker was first coupled, as described above, to peptidyl resins and then, following the removal of the Fmoc protecting group, a Biotin-NHS derivative (6 eq, Aapptec) was attached to the peptidyl resins with triethylamine (TEA, 6 eq) in dimethylformamide. Peptides were then deprotected and removed from the resin *via* an acidolytic treatment with trifluoroacetic acid (TFA) containing 1,2-ethanedithiol (2.5%), phenol (3%) and water (2.5%) for 2 h at room temperature. The diethyl ether-precipitated crude peptides were purified by preparative RP-HPLC performed on a Waters PrepLC 4000 System with a Waters 2487 detector set at 220 nm and an XTerra Prep MS C_18_ column. A linear gradient from eluent A to B with 1% B per 2-min increments (Eluent *A* = H_2_O, 0.1% TFA, Eluent *B* = 60% CH_3_CN/40% H_2_O, 0.1% TFA) was used for each purification. Collected fractions were then analyzed by matrix-assisted laser desorption/ionization – time- of-flight (MALDI-TOF) mass spectrometry (Voyager DE System from Applied Biosystems) in linear mode using the α-cyano-4-hydroxycinnamic acid matrix (Carlsbad, CA, USA) and analytical RP-HPLC with a Phenomenex Jupiter C_18_ column to ensure their homogeneity. Fractions corresponding to the desired product with purity greater than 98% were pooled and lyophilized.

### Production of recombinant hGal-7 and hGal-1

Human Gal-7 cDNA was cloned into pET-22b(+) using *Nde*I and *Hin*dIII restriction enzymes. Human pET-Gal-1 vector was generously donated by Dr. S. Sato (McGill University, QC, Canada). The proteins were produced in *E. coli* BL21 (DE3) cells at 37°C. Isopropyl β-D-1-thiogalactopyranoside (IPTG) (1 mM) was added to the bacterial culture at OD_600nm_ = 0.6–0.7 and then incubated for 4 h at 37°C to allow protein production. Bacterial pellets were resuspended in lysis buffer (0.7 mg/mL lysozyme, 10 mM Tris pH 8, 100 mM NaCl, 1 mM EDTA, 1 mM DTT and a protease inhibitor cocktail) and then incubated for 1 h at 37°C prior to centrifugation for 30 min at 15,000 × *g* (4°C). The supernatant was then filtered with 500 mL bottle top filter (22 μm) (Corning, New York, NY, USA) and then ran through a lactose-agarose column (Sigma, St. Louis, MO, USA). The protein was eluted in 1 mL fractions with 150 mM lactose solution. Purified fractions were analyzed by SDS-PAGE. The hGal-7 was then concentrated and purified using Centrifugal filter units (Amicon Ultra-15, 10K) (EMD, Millipore, Etobicoke, ON) in 20 mM potassium phosphate at pH 7.1. All subsequent experiments with the recombinant proteins were performed in the same buffer solution unless mentioned otherwise. Brilliant Coomassie blue was purchased from BioRad (Bio-Rad Laboratories, Mississauga, ON, Canada). The recombinant protein hGal-2 was purchased from MyBiosource (San Diego, CA, USA).

### Western blotting

For the apoptosis tests, whole-cell extracts were homogenized and resuspended in RIPA buffer (Thermo Fisher Scientific, Rockford, IL, USA) containing protease inhibitors (Roche, Laval, QC, Canada). Equal amounts of whole-cell extracts (25 μg) were separated on SDS-PAGE and transferred onto nitrocellulose membranes (Bio-Rad Laboratories). The membranes were first blocked with 5% milk [w/v] in TBS/0.5% Tween 20 [v/v] for 1 h at room temperature and subsequently blotted overnight in a solution of TBS containing 3% BSA [w/v] and 0.5% Tween 20 [v/v]. The following antibodies were used: a rabbit anti-poly-(ADP-ribose) polymerase (PARP)-1 (p25) polyclonal antibody (1:5000; Epitomics, Burlingame, CA, USA) and a mouse anti-β-actin (1:10000; Sigma-Aldrich, St. Louis, MO, USA). Secondary antibodies consisted of horseradish peroxidase-conjugated donkey anti-rabbit (GE Healthcare, Buckinghamshire, England) and sheep anti-mouse (GE Healthcare) IgG. Detection was performed using the enhanced chemiluminescence method (GE Healthcare). For the recombinant protein tests, each peptide was dissolved and maintained in 20 mM potassium phosphate at pH 7.1. The β-lactose and N-Acetyl-D-lactosamine (LacNAc) powders were purchased from Sigma-Aldrich (St. Louis, MO, USA). The recombinant proteins and peptide dilutions were pre-incubated for 1 h at 4°C prior to gel migration. The native polyacrylamide gel was made without SDS to allow molecular weight differentiation between the dimer and monomer. The following antibodies were used: a goat anti-Gal-7 antibody (1:10000; R&D Systems, Minneapolis, MN, USA), a mouse anti-Gal-1 antibody (1:1000; Proteintech, Chicago, IL, USA) and a rabbit anti-Gal-2 antibody (1:1000; Proteintech, Chicago, IL, USA). Secondary antibodies consisted of donkey anti-goat (R&D Systems) or sheep anti-mouse (GE Healthcare) IgG. Detection was performed as mentioned above.

### Fluorescent binding assay

Recombinant hGal-7 or hGal-1 (10 μg/mL) was coated overnight at 4°C on black, flat bottom, 96-well polystyrene microplates (Ultident, Montreal, QC, Canada). Thereafter, the plate was blocked with Reagent diluent (PBS-BSA 1%) for 1 h, then incubated with unlabeled hGal-7_(129–135)_ (cold) for 60 min and finally incubated with biotin-labeled [Ahx^0^]hGal-7_(129–135)_ for 2 h. Lastly, Streptavidin R-phycoerythrin (1/500, Jackson Immunoresearch, West Grove, PA, USA) was applied to samples for 30 min. All incubations were performed at room temperature and the washes between incubations were done with 20 mM potassium phosphate buffer pH 7.1. The plate was read by a Tecan Infinite M1000 PRO microplate reader at excitation and emission wavelengths of 488 nm and 670 nm, respectively.

### FITC conjugation and hGal-7 binding assay

To assess hGal-7 binding onto Jurkat T cells, 5 μL of a 1.25 mg/mL fluorescein isothiocyanate (FITC)/DMSO solution was added to 300 μL of 1.7 μg/μL recombinant hGal-7 in a 0.1 M NaHCO_3_ pH 9.2 solution and incubated for 1 h at room temperature on a roller. FITC-labeled hGal-7 was then purified using a PD-10 Sepharose column (GE healthcare) and eluted with PBS containing 0.01% [v/v] sodium azide. FITC-labeled hGal-7 (0.1 μM) was then pre-incubated with hGal-7_(129–135)_ (or related peptides) in 20 mM potassium phosphate buffer pH 7.1 for 1 h at 4°C. Jurkat cells (5 × 10^5^ cells per sample) were harvested in PBS-0.01% [v/v] sodium azide and incubated for 30 min on ice with the FITC-labeled hGal-7 with and without peptides. Cells were then washed with PBS-0.01% [v/v] sodium azide and resuspended in 400 μL of the same buffer and analyzed on a FACSCalibur (BD Biosciences).

### Apoptosis assays with Annexin V/PI staining

Apoptosis was measured by flow cytometry using FITC-labeled Annexin V (Biolegend, San Diego, CA, USA) and propidium iodide (PI). Briefly, the corresponding dilutions of recombinant hGal-7 and hGal-7_(129–135)_ peptides were pre-incubated for 1 h at 4°C in serum free RPMI 1640 medium. 2.5 × 10^5^ Jurkat cells were then harvested in the same medium and incubated with their corresponding dilutions at 37°C for 4 h. Cells were washed once in PBS and once in binding buffer (0.01 M HEPES, 0.14 M NaCl, 2.5 mM CaCl_2_, pH 7.4). Cells were then incubated for 15 min with Annexin V in the dark at room temperature. A total of 400 μL of binding buffer containing 0.25 μg/mL propidium iodide was added to the cells before analysis by flow cytometry.

### Statistical analysis

Statistical significance of the experiments was evaluated using the unpaired Student's *t*-test or the Fisher's exact test. Results were considered statistically significant at *P* ≤ 0.05.

## CONCLUSIONS

We have identified a new class of galectin inhibitors that specifically target the dimer interface of hGal-7. Such inhibitor provides an interesting alternative to more conventional galectin inhibitors that target the CRD with soluble glycans. Given the critical role of hGal-7 in cancer, studies are underway to determine whether both types of inhibitors could be used in combination as anti-cancer agents.

## SUPPLEMENTARY FIGURES AND TABLE


