# Supplementary material for: Design of a peptidic inhibitor that targets the dimer interface of a prototypic galectin
Source: Oncotarget. 2015 Oct 1;6(38):40970–80. doi: 10.18632/oncotarget.5403 (PMC4747383; doi:10.18632/oncotarget.5403)
Supplement: Supplementary file 1 [file oncotarget-06-40970-s001.pdf]

SUPPLEMENTARY FIGURES AND TABLE

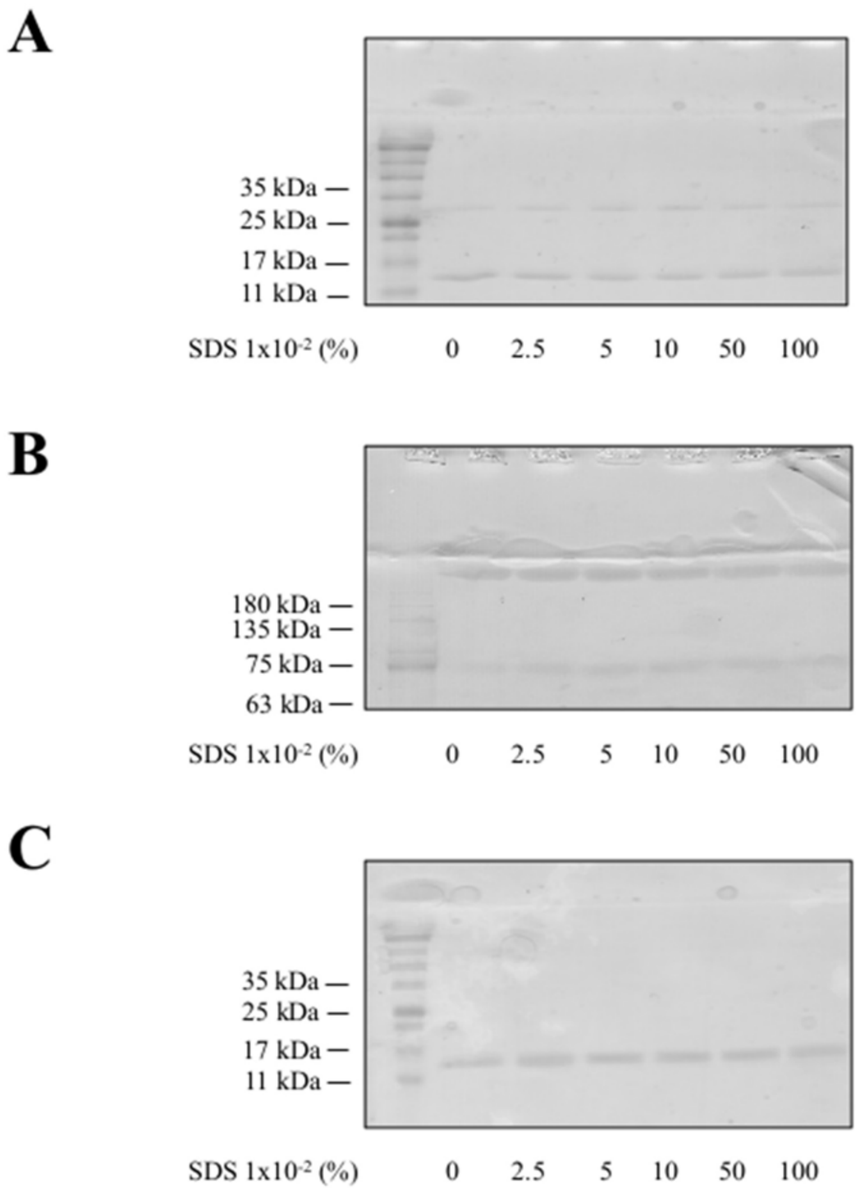

Supplementary Figure S1: The presence of hGaI-7 protein dimer in various (non) denaturing conditions. The recombinant hGaI-7 (1 µM) in the presence of increasing concentrations of SDS was migrated in a polyocrylamide gel containing **A.** no SDS and using 0.1% SDS in the migration buffer, **B.** no SDS and without SDS in the migration buffer and **C.** in a polyacrylamide gel containing 0.1% SDS and 0.1% SDS in the migration buffer after heating 5 min at 95°C with β-mercaptoethanol. In all cases, electrophoresis was carried out for 75 min at 150 V before Coomassie Blue staining.

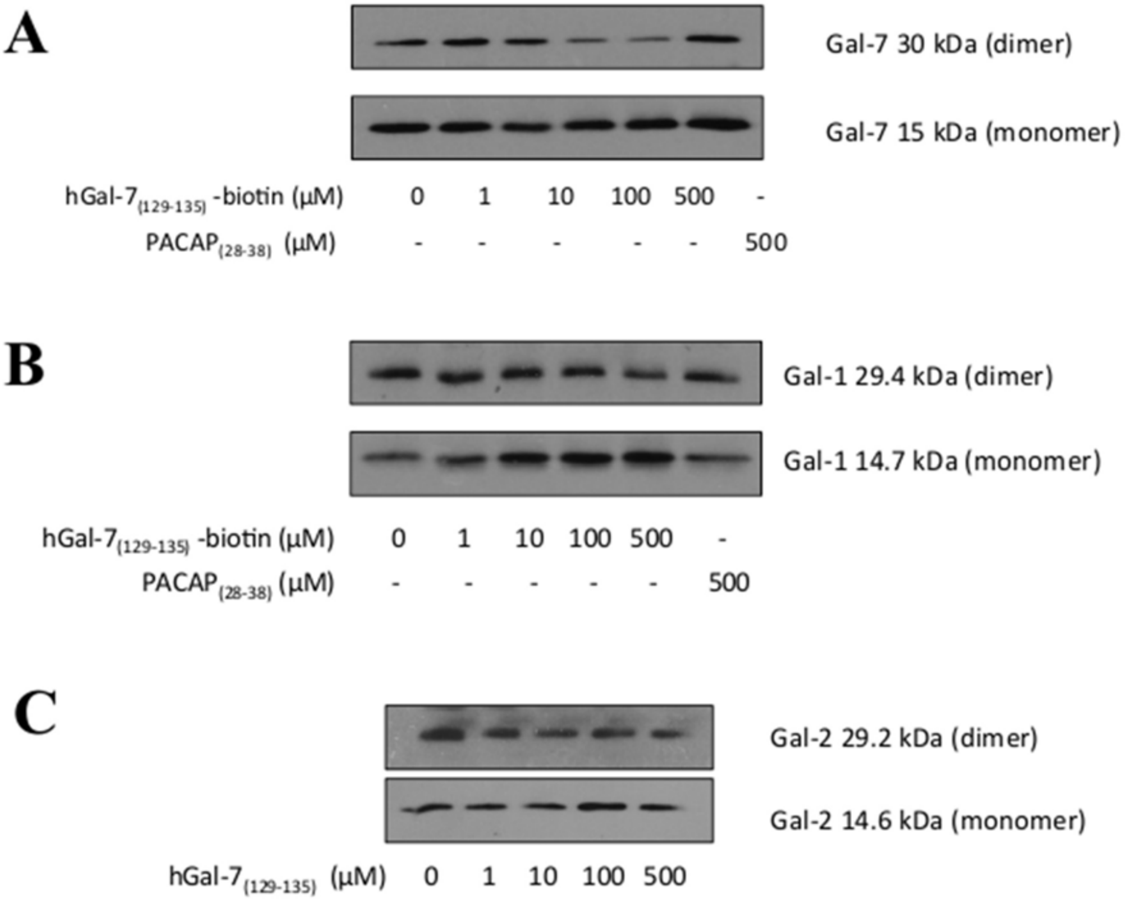

Supplementary Figure S2: Disruption of hGaI-7 dimer by increasing concentrations of hGaI-7$_{129–135}$. A. Recombinant gaI-7 (0.5 µM) was incubated with increasing concentrations of biotin-labeled hGaI-7$_{129–135}$ in 20 mM potassium phosphate buffer (pH 7.1) for 60 min at 4°C beforeWestern blot analysis in low SDS-PAGE using an anti-gaI-7 antibody. **B.** Similar experiments were carried out using recombinant human **B.** gaI-1 and **C.** gaI-2. Results are representative of two independent experiments.

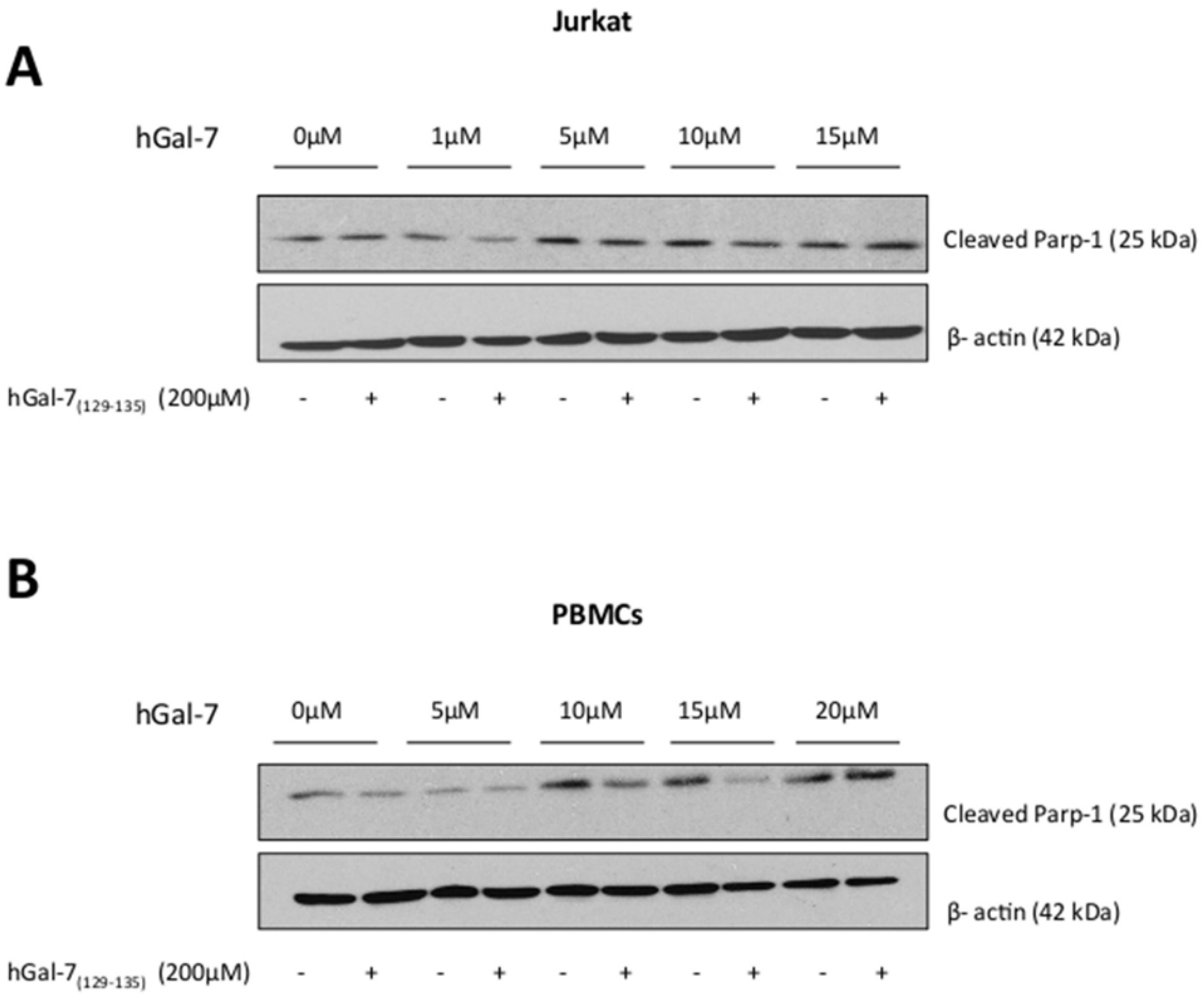

Supplementary Figure S3: hGaI-7$_{129–135}$ decreases gaI-7-induced apoptosis in Jukart T cells and PBMCs. Increasing concentrations of recombinant human gaI-7 were pre-incubated with the hGaI-7$_{129–135}$ (200 μM) prior to its addition to **A.** Jurkat T cells or **B.** PBMCs. Apoptosis wa, measured after 4 h at 37°C in serum·free conditions. Apoptosis by Western blot analysis of parp-1 cleavage. β-actin was used as a loading control. Result are representative of two independent experiments.

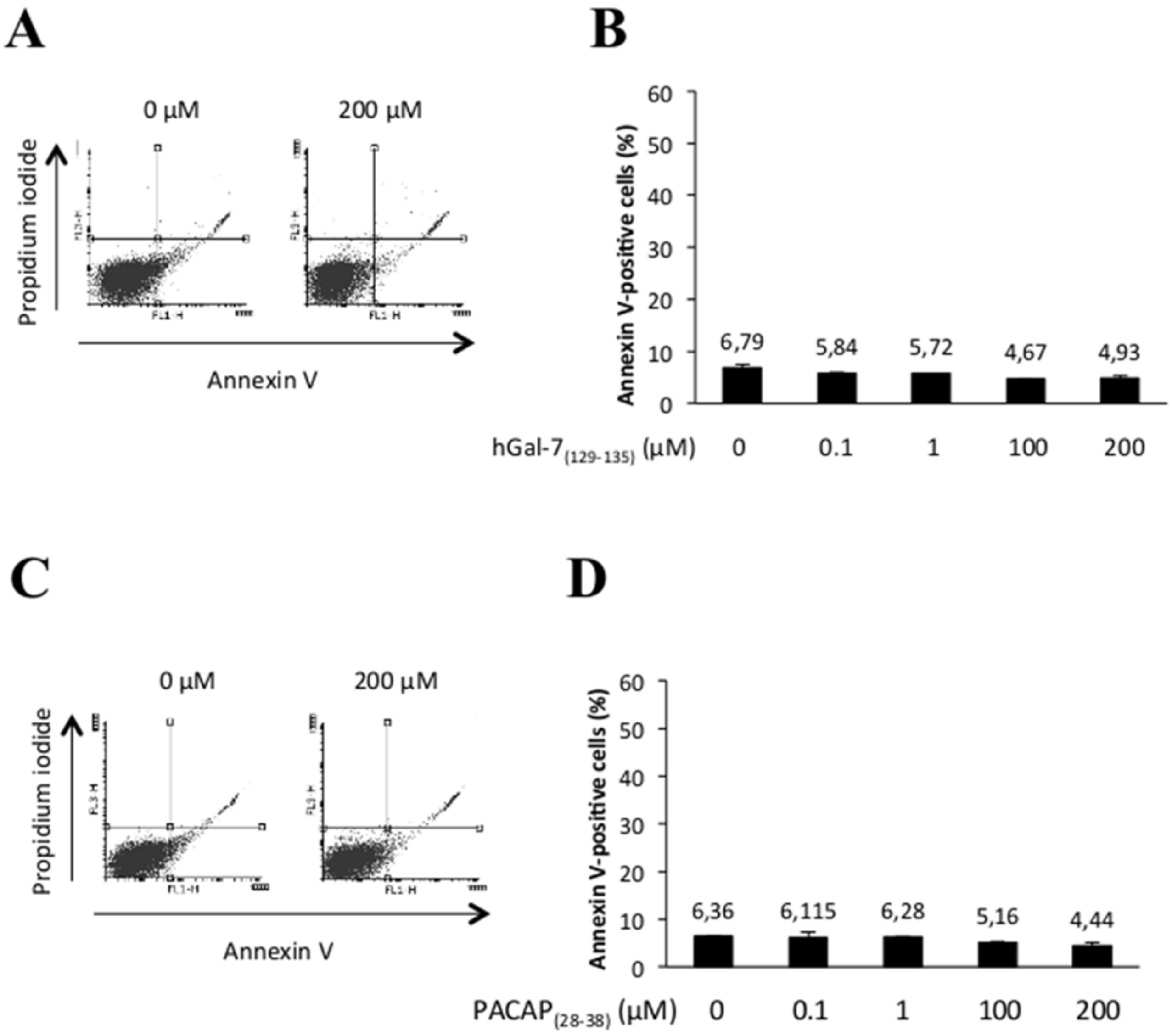

Supplementary Figure S4: Inability of the control peptide (PACAP) to induce apoptosis on Jurkat T cells. Jurkat T cells wee incubated 4 h at 37°C with increasing concentrations of the control peptide PACAP before two-color flow cytometric analysis. *On the left histograms*, cells in the lower right quadrant are representative of annexin V-positive/PI-negative, or early apoptotic cells. Cells in the upper right quadrant indicate annexin V-positive/PI-positive, or late apoptotic cells. The *right histograms* show the percentages of annexin V-positive Jurkat T cells obtained by adding the percentages of cells found in the lower and upper right quadrants. Results are representative of three independent experiments. Error bars represent standard deviation.

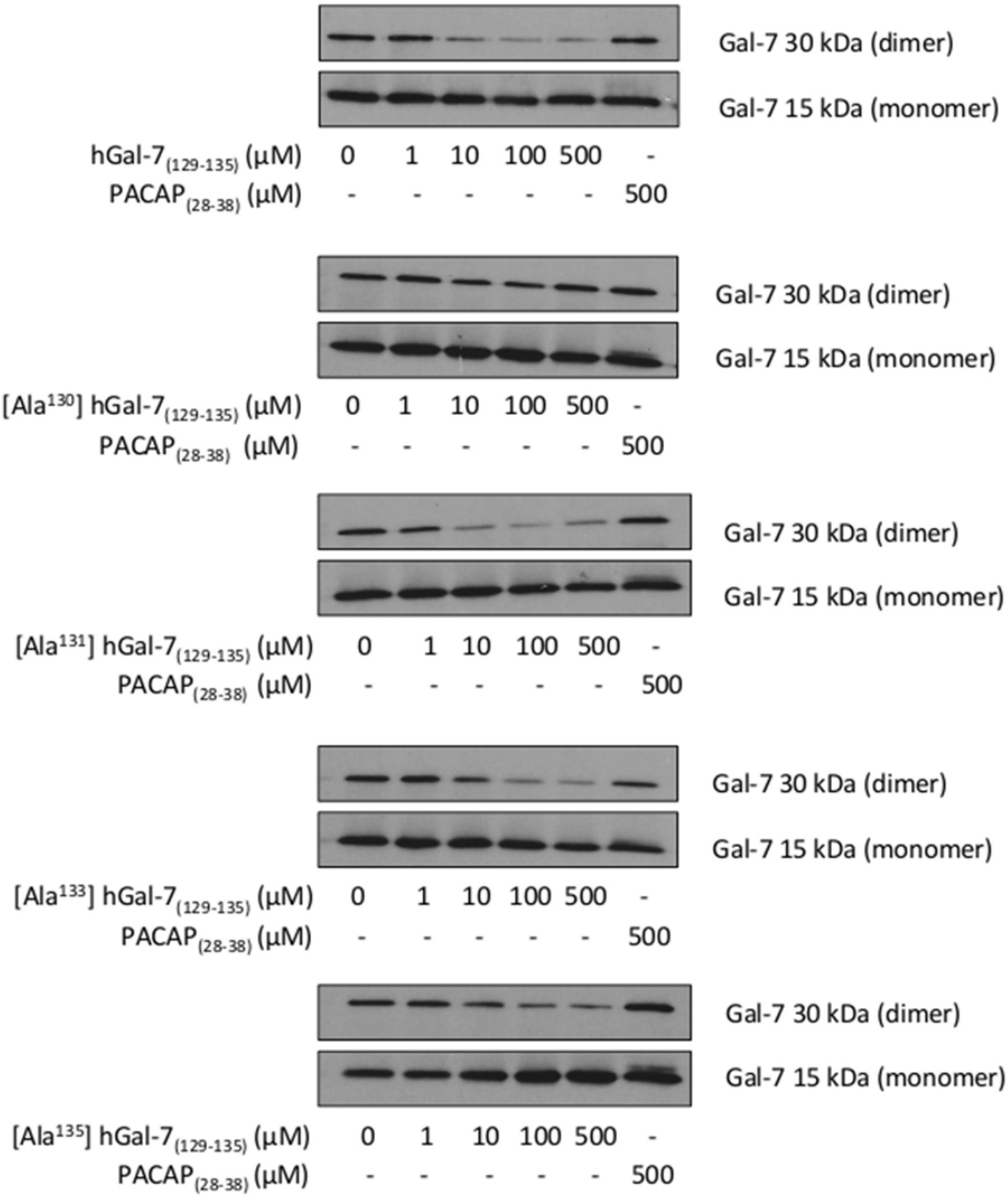

Supplementary Figure S5: Ala-scan of the hGaI-7$_{129–135}$ peptide. Disruption of gaI-7 homodimer (0.5 µM) was measured following addition of increasing concentrations of wild-type or mutant hGaI-7$_{129–135}$ Monomer/homodimer ratios were measured by Western blot analysis using low SDS-PAGE conditions. Results are representative of three independent experiments.

Supplementary Table S1: Overview of hGal-7$_{(129\text{-}135)}$ alanine substitute peptides

Peptide name	Peptide sequence	Theoretical MW (g/mol)	Actual MW (g/mol)
hGal-7 $_{(129\text{-}135)}$	L-D-S-V-R-I-F	849	850.56
[Ala^{130}] hGal-7 $_{(129–135)}$	L-A-S-V-R-I-F	805.10	805.01
[Ala^{131}] hGal-7 $_{(129–135)}$	L-D-A-V-R-I-F	833.02	833.11
[Ala^{133}] hGal-7 $_{(129–135)}$	L-D-S-V-A-I-F	772.92	772.27
[Ala^{135}] hGal-7 $_{(129–135)}$	L-D-S-V-R-I-A	763.90	785.61
